# Pembrolizumab

**DOI:** 10.1186/s40425-015-0078-9

**Published:** 2015-08-18

**Authors:** Leila Khoja, Marcus O. Butler, S. Peter Kang, Scot Ebbinghaus, Anthony M. Joshua

**Affiliations:** Department of Medical Oncology, Princess Margaret Cancer Centre, 610 University Avenue, Toronto, ON M5G 2M9 Canada; Merck & Co., Inc., Kenilworth, NJ USA

**Keywords:** Immune checkpoint blockade, Melanoma, Lung cancer, Pembrolizumab, Programmed death receptor 1, Programmed death receptor ligand 1

## Abstract

The development of the cytotoxic T-lymphocyte-associated protein 4 inhibitor ipilimumab and its approval in 2011 for the treatment of metastatic melanoma has heralded a new era in immuno-oncology. Subsequently, novel agents against the programmed death receptor 1 (PD-1)/programmed death receptor ligand 1 (PD-L1) axis have shown significant activity in melanoma and a variety of other tumor types. Pembrolizumab was the first anti-PD-1 antibody to be approved by the US Food and Drug Administration for the treatment of patients with unresectable or metastatic melanoma with disease progression following ipilimumab, and if *BRAF*^V600^ mutation positive, a BRAF inhibitor. Pembrolizumab has also received breakthrough status for the treatment of *EGFR* mutation-negative, *ALK* rearrangement-negative non-small cell lung cancer (NSCLC) that has progressed on or following platinum-based chemotherapy. There remain a number of pivotal trials in progress to further evaluate the optimal use of pembrolizumab alone and in combination for melanoma, NSCLC, and other tumor types. In this article, we review the efficacy and toxicity profile of pembrolizumab and evaluate its future development.

## Introduction

Discovered in 1992, programmed death receptor 1 (PD-1) is a member of the B7-CD28 superfamily [1]. It is expressed on activated T (CD8^+^ and CD4^+^) cells, B cells, monocytes, natural killer T cells, and antigen-presenting cells (APC), including dendritic cells. Generation of PD-1-deficient mice showed that this receptor has an immune-regulatory role in inducing peripheral tolerance [1–3] and modulating the magnitude of the antigen-specific immune response to infection [4–6] and cancer [7–9]. Inflammation-induced cytokines produced as a result of infection or tumor formation induce the expression of programmed death receptor ligand 1 (PD-L1) on various cell types, including APC, and programmed death receptor ligand 2 (PD-L2) on APC. The PD-1/PD-L1/PD-L2 interaction negatively affects the function of T and B cells, leading to decreased cytokine production and antibody formation, thereby inhibiting autoimmunity and anti-tumor and anti-infectious immunity [10]. Lymphocyte activation relies on antigen recognition by specific T-cell antigen receptors (aided by APCs) and regulation thereafter of that activation by stimulatory and inhibitory signals from T-cell co-receptors. Co-stimulatory receptors include CD28, ICOS, 41BB, and OX40, whilst CTLA-4, VISTA, Tim-3, and PD-1 are co-inhibitory. Dynamic interactions occur between the APC, tumor cell, and T cell that govern whether T-cell activation can occur, and if so, the magnitude and duration of that activity. The role of the various co-stimulatory or co-inhibitory molecules in controlling this interaction is yet to be fully understood, but might well differ within and between patients’ tumor lesions. The role of monocytes or macrophages in this interaction is also under investigation.

The successful development of therapeutic agents targeting the PD-1/PD-L1 axis has been a major therapeutic advancement in oncology. In this article we discuss the development of pembrolizumab, the first anti–PD-1 agent to be approved by the US Food and Drug Administration (FDA). Pembrolizumab is also approved for use in melanoma in Australia, Israel, Korea, Macau, and the United Arab Emirates and was recently recommended for approval in the European Union.

## Review

### Phase I data

**Fig. 1:**
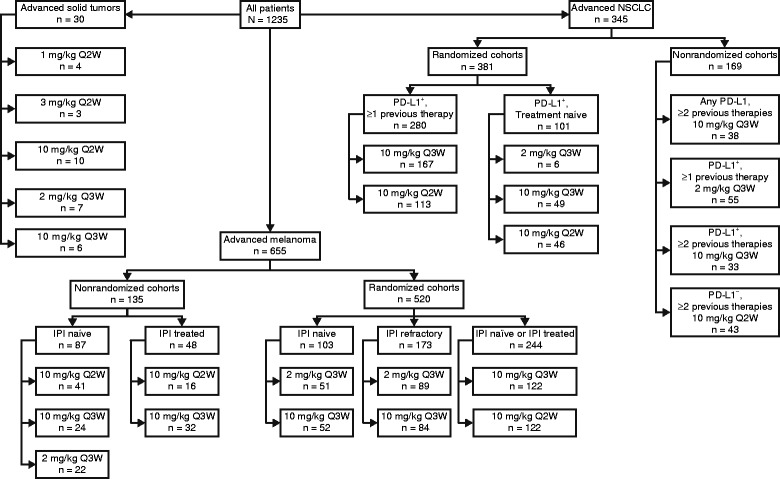
Flowchart summarizing the KEYNOTE-001 treatment cohorts in solid tumors, melanoma, and NSCLC that have been reported to date. Abbreviations: *IPI* ipilimumab; *NSCLC* non-small cell lung cancer; *PD-L1* programmed death receptor ligand 1; *Q2W* once every 2 weeks; *Q3W* once every 3 weeks

Pembrolizumab (previously known as MK-3475 and lambrolizumab) is a potent, highly selective, fully humanized immunoglobulin (Ig) G4-kappa monoclonal antibody against PD-1. The phase I KEYNOTE-001 study (ClinicalTrials.gov identifier: NCT01295827) included the first-in-human dose-finding cohort (part A) that assessed pembrolizumab given intravenously at 1 mg/kg, 2 mg/kg, 3 mg/kg, and 10 mg/kg once every 2 weeks (Q2W) or every 3 weeks (Q3W) [11] and expansion cohorts exploring the safety and antitumor activity of several pembrolizumab doses and schedules in patients with advanced melanoma (parts B and D) and non-small cell lung cancer (NSCLC) (parts C and F) (Fig. 1).

### Melanoma

Based on the first-in-human experience, KEYNOTE-001 was expanded to further explore the safety and efficacy of pembrolizumab in patients with melanoma. Initially, patients with ipilimumab-naive and ipilimumab-treated melanoma were given 10 mg/kg Q2W. Additional cohorts were later recruited to explore pembrolizumab doses and schedules of 2 mg/kg and 10 mg/kg given once every 3 weeks (Q3W) and 10 mg/kg Q2W. One hundred and thirty-five ipilimumab-treated and ipilimumab-naive patients were enrolled in a nonrandomized fashion [12], and 520 patients were enrolled in 1 of 3 randomized cohorts: i) 2 or 10 mg/kg Q3W for ipilimumab-refractory disease [13], ii) 2 or 10 mg/kg Q3W for ipilimumab-naive disease [14], and iii) 10 mg/kg Q2W or Q3W for ipilimumab-treated or ipilimumab-naive disease [15] (Fig. 1). In all cohorts, patients were required to have an Eastern Cooperative Oncology Group performance status of 0 or 1 and stable brain metastases for at least 8 weeks (brain magnetic resonance imaging [MRI] scans were not required during screening). Tumor imaging was performed at 12-week intervals.

The first data to be reported for pembrolizumab in melanoma were from patients enrolled in a nonrandomized manner (analysis cutoff date, February 2013) [12]. For these patients, ipilimumab-treated disease was defined as progression within 6 months after the first dose of ipilimumab, and ≤3 prior treatments were allowed [16]. Up to 2 previous treatments were allowed for all ipilimumab-naive patients [17]. Prior treatment with BRAF inhibitors was not mandatory for patients with *BRAF*-mutant tumors [16]. Of the 135 nonrandomized patients, 117 were evaluable by Response Evaluation Criteria in Solid Tumors version 1.1 (RECIST v1.1) per independent central review, and all 135 patients were evaluable by immune-related response criteria (irRC) [18] per investigator review [12]. The confirmed overall response rate (ORR) across all doses was 38 % by RECIST v1.1 and 37 % by irRC. No significant effect of prior ipilimumab treatment was seen on response rates (per RECIST v1.1, 38 % for ipilimumab-treated patients, 37 % for ipilimumab-naive patients) [12].

Nonrandomized data revealed numerical differences in ORR between doses and schedules, although the 95 % confidence intervals (CIs) largely overlapped; ORR was 52 % (95 % CI: 38–66) at 10 mg/kg Q2W (n = 52), 27 % (95 % CI: 15–42) at 10 mg/kg Q3W (n = 45), and 25 % (95 % CI: 9–49) at 2 mg/kg Q3W (n = 20) [12]. Of the 52 patients with confirmed or unconfirmed response, 42 remained on treatment after a median follow-up duration of 11 months. Best overall response was complete response (CR) in 13 patients (11 %) and partial response (PR) in 39 patients (33 %). Of note, 17 (33 %) of the 52 responders experienced stable disease (SD) at an early response assessment, but went on to have durable responses with continued treatment [12]. Similar to the delayed or atypical responses observed with ipilimumab [18], a comparison of response per RECIST v1.1 and irRC in the melanoma cohorts of KEYNOTE-001 revealed unique response patterns with pembrolizumab; 7 patients (3.4 %) had early pseudoprogression and 8 patients (3.9 %) had delayed pseudoprogression [19]. Further validation of irRC is ongoing, but the patterns of response observed to date may have implications for optimal management for patients on anti-PD-1 agents [19].

After an additional period of follow-up (analysis cutoff date, October 2013), the confirmed ORR by RECIST v1.1 was 41 %, 87 % of responses were ongoing, and an initial CR was observed after as many as 62 weeks of treatment [17]. In a combined analysis across doses, median progression-free survival (PFS) was 7 months. Based on a cutoff date of May 2014, median overall survival (OS) was not reached, and estimated OS at 12 and 18 months was 81 % and 71 %, respectively [17]. The highest rate of treatment-related adverse events (AEs) was seen at 10 mg/kg Q2W (23 %, compared with 4 % at 10 mg/kg Q3W and 9 % at 2 mg/kg Q3W) [17]. The higher rate of AEs at the 10 mg/kg Q2W dose may be due to the higher amount of drug delivered over time, but may also be secondary to a longer follow-up duration or potentially, a reflection of the Q2W schedule, which provides patients with more opportunities to report AEs. Treatment was deemed to be safe at all doses, with 13 % of patients experiencing grade 3/4 toxicities [12].

One of the randomized cohorts of KEYNOTE-001 included 173 ipilimumab-refractory patients who were randomly assigned to pembrolizumab 2 mg/kg Q3W (n = 89) or 10 mg/kg Q3W (n = 84) (Fig. 1) [13]. The patient population was heavily pretreated, with any number of prior therapies allowed. Seventeen percent of patients were *BRAF* mutation positive (pretreatment with targeted therapy was mandatory), and 39 % had elevated lactate dehydrogenase (LDH) levels [13]. Ipilimumab-refractory disease was defined as confirmed progression within 6 months after the last dose of ipilimumab, with ≥2 doses of ipilimumab required [16]. At the time of reporting (analysis cutoff date, October 2013), the median follow-up duration was 8 months [13]. ORR at both doses was 26 % per RECIST v1.1 by central review (*P* = 0.96), and 42 % of patients remained on treatment [13]. The median time to response was 12 weeks (range 7–36 weeks); 1 patient in each arm had a CR, 25 % in each arm had PR, and SD was observed in 25 % (2 mg/kg) and 24 % (10 mg/kg). Median PFS was 22 weeks in the 2-mg/kg group and 14 weeks in the 10-mg/kg group (hazard ratio [HR] = 0.84; 95 % CI: 0.57–1.23), with 24-week PFS rates of 45 % and 37 %, respectively [13]. As of May 2014, median OS was not reached; 1-year OS rates were 58 % at 2 mg/kg and 63 % at 10 mg/kg (HR = 1.09; 95 % CI: 0.68–1.75) [13]. Eighty-two percent of patients in each arm experienced a treatment-related AE; the occurrence of treatment-related grade 3/4 toxicities was 12 % overall, with only 5 % reporting a serious treatment-related AE. There was no significant difference in the rate of AEs between the 2 arms. Treatment discontinuation due to treatment-related toxicity of any grade occurred in 3 % of patients (6 % in the 2-mg/kg group and 1 % in the 10-mg/kg group) [13].

In the ipilimumab-naive randomized cohort, 103 patients were randomly assigned to receive pembrolizumab 2 mg/kg (n = 51) or 10 mg/kg (n = 52) Q3W [14]. Of these patients, 27 %–41 % had elevated LDH, 49 %–56 % had no prior treatment, and 31 %–39 % were *BRAF* mutant (prior BRAF-inhibitor treatment was not required in this cohort). After 12 months of follow-up (analysis cutoff date, October 2013), ORR per RECIST v1.1 was 33 % in the 2-mg/kg group and 40 % in the 10-mg/kg group (*P* = 0.48), with 4 CRs in each arm [14]. Median PFS was 27 weeks and 23 weeks, with 24-week PFS rates of 50 % and 48 %, respectively. 1-year OS rates were 72 % at 2 mg/kg and 64 % at 10 mg/kg. Median time to response was 12 weeks (i.e., the time of the first tumor assessment) for both doses. Treatment was well tolerated, and although treatment-related AEs occurred in approximately 86 % of patients, the incidence of grade 3 or greater toxicities was 22 % in the 2-mg/kg group and 2 % in the 10-mg/kg group. Two percent of patients discontinued treatment in each arm because of an AE, but these were not deemed to be treatment related [14].

The final randomized melanoma cohort of KEYNOTE-001 included 244 patients with ipilimumab-naive or ipilimumab-treated disease (defined as above) who were randomly assigned to pembrolizumab 10 mg/kg Q2W (n = 123) or Q3W (n = 121) to further explore response and outcome at these schedules [15]. The primary outcome was ORR by RECIST v1.1 determined by central review. As of the April 2014 analysis cutoff date, a total of 224 patients were evaluable for response (n =107 at Q3W and n =117 at Q2W). ORR was 31 % in the Q3W arm and 35 % in the Q2W arm, indicating no difference in ORR between schedules (*P* = 0.5052). With Q3W dosing, 3 CRs and 28 PRs were seen, whilst on Q2W dosing, 6 CRs and 29 PRs were observed [15]. ORR per RECIST v1.1 was 37 % in the ipilimumab-naive patients (n = 113) and 30 % in the ipilimumab-treated patients (n = 111). PFS was similar on both schedules (HR = 1.19; 95 % CI: 0.86–1.64). Treatment-related AEs were experienced in 82 % of Q3W and 81 % of Q2W patients, while treatment-related grade 3/4 toxicities were observed in 12 % and 15 %, respectively. Treatment-related AEs leading to discontinuation occurred in 1 % of Q2W patients and 3 % of Q2W patients [15].

Taken together, data from KEYNOTE-001 show significant activity for pembrolizumab in patients with advanced melanoma, regardless of prior ipilimumab treatment. Patients with ipilimumab-naive disease appear to have higher response rates. Notably, data from randomized studies do not show a statistically significant difference in activity between doses and schedules. The majority of responses in all cohorts were observed at the time of the first imaging assessment at week 12, and although delayed response after initial SD or progressive disease was possible, it was rare.

Based on the data obtained from the randomized cohort of 173 ipilimumab-refractory patients and supportive data from other KEYNOTE-001 melanoma cohorts, pembrolizumab 2 mg/kg Q3W is now FDA approved for the treatment of patients with unresectable or metastatic melanoma with disease progression following ipilimumab, and if *BRAF*^V600^mutation positive, a BRAF inhibitor. The KEYNOTE-002 trial (ClinicalTrials.gov identifier: NCT01704287), a randomized, controlled study in which 2 doses of pembrolizumab (2 mg/kg [n = 180] or 10 mg/kg Q3W [n = 181]) were compared with investigator-choice chemotherapy (n = 179), confirmed the efficacy and safety of pembrolizumab in this population [20]. Eighty-three percent of enrolled patients had M1c disease and 73 % received ≥2 lines of prior therapy including ipilimumab. The 2 co-primary endpoints are PFS and OS, with ORR as a secondary endpoint; cross over to pembrolizumab is allowed upon progression on chemotherapy. A prespecified interim analysis (conducted after ≥270 PFS events at a 0.25 % significance level) after a median follow-up time of 10 months showed 6-month PFS (by RECIST v1.1) of 34 % (95 % CI: 27 %–41 %) for 2 mg/kg, 38 % (95 % CI: 31 %–45 %) for 10 mg/kg, and 16 % (95 % CI: 10 %–22 %) for the chemotherapy group (HR = 0.57 for 2 mg/kg, 0.5 for 10 mg/kg; *P* = 0.0001 for either arm compared with chemotherapy). No significant differences in PFS were found between the 2 doses of pembrolizumab (HR = 0.91 [range, 0.71–1.16]; *P* = 0.44). Data on OS are awaited. KEYNOTE-030 (ClinicalTrials.gov identifier: NCT02083484), an expanded access program using the approved 2 mg/kg Q3W regimen, is open outside of the United States for patients with ipilimumab-refractory melanoma.

Other studies of pembrolizumab in melanoma are ongoing. KEYNOTE-006 (ClinicalTrials.gov identifier: NCT01866319), a registration trial in which pembrolizumab 10 mg/kg Q2W (n = 279) and 10 mg/kg Q3W (n = 277) given for 2 years are being compared with ipilimumab (n = 278) in patients with ipilimumab-naive melanoma, has reported data from 2 preplanned interim analyses for the 2 co-primary endpoints of PFS and OS (based on intention to treat) [21]. The first analysis was performed for PFS after ≥260 events and at least 6 months of follow-up. The second analysis occurred for OS after ≥290 deaths in all study groups and at least 9 months or a minimum of 12 months follow-up had occurred (analysis cutoff date, March 3, 2015; median duration of follow-up 7.9 months [range, 6.1–11.5]) [21]. At baseline, LDH level was elevated in 32.4 % of patients, 65.3 % of patients had stage M1c disease, 80.5 % had PD-L1–positive tumors, 36.2 % had *BRAF*^V600^ mutant tumors, and 9.4 % had stable brain metastases; 65.8 % of patients had received no prior therapy for advanced disease. At the first interim analysis 502 events had occurred and PFS was significantly higher for both doses of pembrolizumab compared with ipilimumab; estimated 6-month PFS was 47.3 % (Q2W schedule), 46.4 % (Q3W) and 26.5 % (ipilimumab) [21]. Median PFS was 5.5 months (95 % CI: 3.4–6.9) for pembrolizumab Q2W, 4.1 months (95 % CI: 2.9–6.9) for pembrolizumab Q3W, and 2.8 months (95 % CI: 2.8–2.9) for ipilimumab. HRs for the pembrolizumab groups compared with ipilimumab were 0.58 (95 % CI: 0.46–0.72; *P* < 0.001) and 0.58 (95 % CI: 0.47–0.72; *P* < 0.001) for Q2W and Q3W schedules, respectively [21]. There was no difference in PFS in any subgroup with any of the 3 treatments. At the second interim analysis, 289 deaths had occurred, and OS estimates at 1 year were 74.1 %, 68.4 %, and 58.2 % for pembrolizumab Q2W, pembrolizumab Q3W, and ipilimumab, respectively. Compared with the ipilimumab group, HR was 0.63 (95 % CI: 0.47–0.83; *P* < 0.0005) in the Q2W arm and 0.69 (95 % CI: 0.52–0.9; *P* = 0.0036) in the Q3W arm [21]. Median OS has not been reached in any group. OS was similar across subgroups, with the exception of PD-L1 expression. For PD-L1-negative tumors, HR was 0.91 in the Q2W arm and 1.02 in the Q3W group, compared with ipilimumab. ORR was significantly higher in the pembrolizumab arms (33.7 % for Q2W, 32.9 % for Q3W; *P* < 0.001 for either schedule) compared with ipilimumab (11.9 %) [21]. Complete responses were seen in 5.0 %, 6.1 %, and 1.4 %, respectively. Median duration of response was not achieved in any group. Median time to response was 86 days (range, 32–212), 85 days (range, 36–251), and 87 days (range, 80–250), respectively, and over 88 % of responses in all groups were ongoing at the time of analysis [21]. The data and safety monitoring committee has recommended that pembrolizumab be made available to patients who have progressed in the ipilimumab group. Follow-up for safety and survival will continue until final analysis [21].

Additional trials of pembrolizumab in melanoma are exploring the potential to treat asymptomatic brain disease (NCT02085070) and the activity and safety of combination therapy with pegylated interferon alpha (NCT02112032 and NCT02089685 [KEYNOTE-029]), dabrafenib and trametinib (KEYNOTE-022, NCT02130466), and ipilimumab (KEYNOTE-029, NCT02089685).

### Lung carcinoma

Results from the NSCLC cohort of KEYNOTE-001 (Fig. 1) have been recently reported [22]. A total of 495 patients were enrolled and received ≥1 cycle of pembrolizumab. Patients received either 2 mg/kg Q3W (n = 6), or 10 mg/kg Q2W (n = 202) or Q3W (n = 287) (Fig. 1) and response was assessed per RECIST v1.1. Across doses, schedules, and degrees of PD-L1 expression, ORR was 19.4 % (95 % CI: 16.0–23.2) [22]. ORR was 24.8 % (95 % CI: 16.7–34.3) in treatment-naive patients (n = 101) and 18.0 % (95 % CI: 14.4–22.2) in treatment-naive patients (n = 101), and 18.0 % (95 % CI: 14.4–22.2) in previously treated patients (n = 394). There was no difference in response according to histology, dose, or schedule [22]. Pembrolizumab demonstrated an ORR of 33.3 % (95 % CI: 4.3–77.7) at 2 mg/kg Q3W (n = 6), 19.2 % (95 % CI: 14.8–24.2) at 10 mg/kg Q3W (n = 287), and 19.3 % (95 % CI: 14.1–25.4) at 10 mg/kg Q2W (n = 202) [22]. Previous or current smokers had an ORR of 22.5 % compared with 10.3 % in nonsmokers. The median duration of response at the time of analysis (August 2014) was 12.5 months (range, 1.0–10.4) in all patients, 10.4 months (range, 1–10.4) in previously treated patients, and 23.3 months (range, 1–23.3) in treatment-naive patients. Survival analysis showed a median PFS of 3.7 months (95 % CI: 2.9–4.1) for all patients (3 months for previously treated patients [95 % CI: 2.2–4.0] and 6 months [95 % CI: 4.1–8.6] for treatment-naive patients). Median OS was 12 months (95 % CI: 9.3–14.7) for all patients (9.3 months for previously treated patients [95 % CI: 8.4–12.4] and 16.2 months [95 % CI: 16.2–not reached] for treatment-naive patients) [22]. As presented in more detail below in the “Biomarkers of Response” section, there was a relationship between degree of PD-L1 expression and outcomes such that patients with PD-L1 expression in ≥50 % of tumor cells had higher ORR and longer PFS and OS compared with patients who had PD-L1 expression in <50 % of tumor cells. Treatment was well tolerated, with 9.5 % of patients overall experiencing grade ≥3 treatment-related AEs, most commonly pneumonitis (1.8 %) [22].

Several trials in patients with NSCLC are ongoing to evaluate pembrolizumab as a single agent or in combination as first-line or second-line treatment. KEYNOTE-042 (NCT02220894) and KEYNOTE-024 (NCT02142738) are randomized phase III studies of patients with treatment-naive, PD-L1-positive advanced or metastatic NSCLC without sensitizing *EGFR* mutation or *ALK* translocation. Patients are being randomized to either pembrolizumab 200 mg Q3W or platinum-containing chemotherapy. A total of 1240 patients will be recruited to KEYNOTE-042 and 300 will be enrolled in KEYNOTE-024, with OS and PFS as the primary end points, respectively. KEYNOTE-010 (NCT01905657) will evaluate pembrolizumab as second-line therapy in comparison with docetaxel following progression on platinum-based chemotherapy in 920 patients. In KEYNOTE-021 (NCT02039674), 320 patients will be recruited in a 2-part, multiarm study. Part 1 will determine the optimal combination of pembrolizumab with chemotherapy, targeted agents, or ipilimumab; part 2 will compare pembrolizumab in combination with chemotherapy to chemotherapy alone, and will assess the optimal combination of ipilimumab and pembrolizumab. KEYNOTE-037 (NCT02178722) is designed to test the combination of pembrolizumab and the indoleamine 2,3-dioxygenase (IDO1) inhibitor INCB024360. Part 1 will be a phase I study in advanced solid tumors to determine the optimal phase II dose of INCB024360 in combination with pembrolizumab 2 mg/kg Q3W. Part 2 will test the combination in NSCLC in a randomized, double-blind, phase II study.

### Additional tumor types

Pembrolizumab has demonstrated efficacy in other advanced solid tumors and hematologic malignancies. KEYNOTE-012, which recruited patients with ≥1 % PD-L1 tumor cell positivity to receive pembrolizumab 10 mg/kg Q2W for up to 24 months, included head and neck cancer [23], gastric carcinoma [24], urothelial carcinoma [25], and triple-negative breast cancer [26] (Table 1). In gastric carcinoma, 40 % (65/162) of screened patients were PD-L1 positive [24]. Thirty-nine patients with gastric cancer were enrolled (19 from Asia and 20 from the rest of the world) and treated with pembrolizumab; 67 % had received ≥2 prior therapies and median follow-up was 8.8 months at the time of reporting. ORR was 22 % (95 % CI: 10–39), 6-month PFS was 24 %, and 6-month OS was 69 % [24]. In urothelial carcinomas, 33 patients were treated, 52 % of whom had received ≥2 previous treatments [25]. ORR was 25 % (95 % CI: 10–44), median PFS was 2 months, and median OS was 12.7 months [25]. Patients with triple-negative breast cancer enrolled in KEYNOTE-012 had an ORR of 18.5 % (5/27 evaluable patients), 7 patients had SD, and median PFS was 1.9 months [26]. Of the 104 patients with head and neck cancer who were screened, 81 (78 %) were PD-L1 positive (23 human papillomavirus [HPV] positive and 35 HPV negative) [23]. ORR was 20 % regardless of HPV status [23]. In KEYNOTE-013, ORR was 66 % in patients with Hodgkin lymphoma [27] (Table 1). More recently, preliminary data from the mesothelioma cohort of the KEYNOTE-028 study of pembrolizumab for advanced solid tumors showed ORR in 28 % (7/25) of patients, including SD in 48 % (12/25) [28].

**Table 1:** Summary of pembrolizumab efficacy and safety in advanced malignancies other than melanoma and NSCLC

Study (clinical trials.gov identifier)	N	Study design	Pembrolizumab dose/schedule	Efficacy (RECIST v1.1, central review)	Safety
HNSCC					
KEYNOTE-012	60	Phase I, international, open-label, nonrandomized cohort of PD-L1-positive advanced HNSCC	10 mg/kg Q2W	• ORR: 20 %	• Grade 3-4 DRAEs: 17 %
(NCT01848834) [23]					
				• Median duration of response: NR (range 8+ to 41+ weeks)	• DR discontinuations: not reported
				• Median PFS: 9.3 weeks (95 % CI: 8.0–20.1)	• DR deaths: none
				• Median OS: 12.6 months	
				• 6-month OS rate: 65 %	
Gastric cancer					
KEYNOTE-012	39	Phase I, international, open-label, nonrandomized cohort of PD-L1-positive advanced gastric cancer	10 mg/kg Q2W	• ORR: 22 %	• Grade 3-4 DRAEs: 10 %
(NCT01848834) [24]					
				• Median duration of response: 24 weeks (range 8+ to 33+ weeks)	• DR discontinuations: none
				• Median PFS: 1.9 months (95 % CI: 1.8–3.5)	• DR deaths: n = 1
				• Median OS: NR	
				• 6-month OS rate: 69 %	
Urothelial cancer					
KEYNOTE-012	33	Phase I, international, open-label, nonrandomized cohort of PD-L1-positive advanced urothelial cancer	10 mg/kg Q2W	• ORR: 25 %	• Grade 3-4 DRAEs: 15 %
(NCT01848834) [25]					
				• Median duration of response: NR (range 16 to 50+ weeks)	• DR discontinuations: 3 %
				• Median PFS: 2 months (95 % CI: 1.7–4.0)	• DR deaths: none
				• Median OS: 9.3 months	
				• 12-month OS rate: 55 %	
Triple-negative breast cancer					
KEYNOTE-012	32	Phase I, international, open-label, nonrandomized cohort of PD-L1-positive advanced triple-negative breast cancer	10 mg/kg Q2W	• ORR: 19 %	• Grade 3-4 DRAEs: 16 %
(NCT01848834) [26]					
				• Median duration of response: NR (range 15 to 40+ weeks)	• DR discontinuations: 3 %
				• Median PFS: 1.9 months (95 % CI: 1.7–5.4)	• DR deaths: n = 1
				• OS: not reported	
Hodgkin lymphoma					
KEYNOTE-013	29	Phase I, international, open-label, nonrandomized cohort of PD-L1-positive Hodgkin lymphoma	10 mg/kg Q2W	• ORR: 66 %	• Grade 3-4 DRAEs: 10 %
(NCT01953692) [27]					
				• Median duration of response: NR (range 1+ to 185+ days)	• DR discontinuations: not reported
				• PFS: not reported	• DR deaths: n = 0
				• OS: not reported	

Clinical data reported to date

Abbreviations: *AE* adverse event; *CI* confidence interval; *DR* drug-related; *DRAE* drug-related AE; *HNSCC* head and neck squamous cell carcinoma; *NR* not reached; *NSCLC* non-small cell lung cancer; *ORR* overall response rate; *OS* overall survival; *PD-L1* programmed death receptor ligand 1; *PFS* progression-free survival; *Q2W* once every 2 weeks; *Q3W* once every 3 weeks

### Toxicity

Toxicity reported to date, primarily in melanoma patients, has been manageable and not treatment limiting in the majority of patients. The most common AEs have been fatigue, rash, pruritus, arthralgia, amylase elevation, and diarrhea. Other toxicities such as nephritis or colitis are rare and there have also been case reports of individual idiosyncratic reactions, including diabetes and heart failure [29, 30]. AEs are generally immune-related but manageable with corticosteroids and interruptions of dosing. The specific management of immune-related AEs has followed guidelines drawn from previous experience with ipilimumab; to date, a number of treatment guidelines exist [31, 32].

Common AEs of any grade detected in patients enrolled in KEYNOTE-001 are described in Table 2. Few grade 3 or above AEs have been seen; however, the most prevalent are pneumonitis, diarrhea, hepatitis, and endocrine-related AEs such as hyper- or hypothyroidism. Grade 3 or above AEs reported in patients treated in KEYNOTE-001 are described in Table 3. The incidence of grade ≥3 AEs was 14 % in the nonrandomized melanoma cohorts (n = 135) [17] and 12 % in the randomly assigned ipilimumab-refractory patients (n = 173) [13]. In KEYNOTE-006 patients with melanoma, the pembrolizumab arms had less toxicity compared with ipilimumab; incidence of grade ≥3 toxicity was 13.3 %, 10.1 %, and 19.9 % for the 10 mg/kg Q2W vs 10 mg/kg Q3W doses of pembrolizumab and ipilimumab, respectively [21]. The rate of drug discontinuation secondary to AEs for these groups was 4.0 %, 6.9 %, and 9.4 %, respectively. Common AEs observed with pembrolizumab were fatigue, diarrhea, hyperthyroidism, hypothyroidism, rash, and pruritus; grade ≥3 diarrhea occurred in >1 % of patients (2.5 % and 1.1 %, respectively, for Q2W vs Q3W schedules) [21]. Grade ≥3 colitis occurred in 1.4 % and 2.5 % and grade ≥3 hepatitis in 1.1 % and 1.8 % at the Q2W and Q3W schedules, respectively, detailed in Tables 2 and 3 [21].

**Table 2:** DRAEs with incidence ≥5 % observed in patients from KEYNOTE-001 and KEYNOTE-006

AE, %	Nonrandomized and randomized cohorts KEYNOTE-001	NSCLC cohorts KEYNOTE-001	KEYNOTE-006	KEYNOTE-006
	(n = 411) [16]	(n = 495) [22]	(melanoma, 10 mg/kg Q2W, n = 278) [21]	(melanoma, 10 mg/kg Q3W, n = 277) [21]
Fatigue	36	19	21	19
Pruritus	24	11	14	14
Rash	20	10	15	13
Arthralgia	16	9	9	12
Diarrhea	16	8	17	14
Nausea	12	8	10	11
Vitiligo	11	NR	9	11
Asthenia	9	5	12	11
Cough	9	2	4	4
Myalgia	9	3	7	2
Headache	8	2	3	2
Hypothyroidism	8	7	10	9
Decreased appetite	7	11	6	7
Dyspnea	7	4	1	3
Chills	6	2	1	0
Pyrexia	6	4	4	1
ALT increase	5	2	4	1
Pneumonitis	3	4	<1	2
Hyperthyroidism	1	2	7	3
Colitis	<1	NR	2	4
Hepatitis	<1	NR	1	2
Hypophysitis	NR	NR	<1	<1
Nephritis	NR	NR	0	<1

KEYNOTE-001 included melanoma and lung cohorts; KEYNOTE-006 included patients with melanoma

Numbers given as percentages where available

Abbreviations: *AE* adverse event; *ALT* alanine aminotransferase; *DRAEs* drug-related AEs; *NR* not reported; *NSCLC* non-small cell lung cancer

**Table 3:** Incidence of grade ≥ 3 DRAEs in patients from KEYNOTE-001 and KEYNOTE-006

AE, %	Nonrandomized and randomized cohorts	NSCLC cohorts	KEYNOTE-006	KEYNOTE- 006
	(n = 411) [16]	(n = 495) [22]	(melanoma, 10 mg/kg Q2W, n = 278) [21]	(melanoma,10 mg/kg Q3W, n = 277) [21]
Fatigue	2	<1	0	<1
ALT increase	<1	<1	0	<1
Colitis	<1	NR	1	3
Decreased appetite	<1	1	0	0
Diarrhea	<1	<1	3	1
Dyspnea	<1	4	0	<1
Headache	<1	NR	0	0
Hepatitis	<1	NR	1	2
Hyperthyroidism	<1	NR	0	0
Hypophysitis	<1	NR	<1	<1
Hypothyroidism	<1	<1	<1	0
Nausea	<1	<1	0	<1
Pneumonitis	<1	2	0	<1
Pruritus	<1	0	0	0
Rash	<1	<1	0	0
Arthralgia	0	<1	0	<1
Asthenia	0	1	<1	0

KEYNOTE-001 included melanoma and lung cohorts; KEYNOTE-006 included patients with melanoma

Numbers given as percentages where available

Abbreviations: *AE* adverse event; *ALT* alanine aminotransferase; *DRAE* drug-related AEs; *NR* not reported; *NSCLC* non-small cell lung cancer

Lung patients treated on KEYNOTE-001 had a similar safety profile to that observed in patients with melanoma. The incidence of AEs of grade ≥3 was 9.5 %, with pneumonitis (1.8 %), dyspnea (3.8 %), decreased appetite (1 %), and asthenia (1 %) having the highest frequencies [22]. Pneumonitis of any grade occurred in 3.6 % of patients [22]. To date, no specific associations in patients with NSCLC have been reported between the risk of pneumonitis and previous or subsequent radiotherapy after progression on an anti-PD-1/PD-L1 agent. There was 1 treatment-related death (pneumonitis) [22].

Median follow-up duration in all reported and published trial cohorts has been short, and mature data on long-term toxicity are awaited. The kinetics of toxicity will be important in managing patients on pembrolizumab, especially in patients undergoing continued treatment within the context of durable CR or PR. Important issues for future investigation include how the profile of AEs will alter with combinatorial immune or multimodality therapies, such as vaccines and radiation therapy.

### Biomarkers of response

The first phase I trials to demonstrate safety and activity of agents targeting the PD-1/PD-L1 axis evaluated nivolumab [33] and BMS-936559 [34], respectively. Immunohistochemical (IHC) staining for PD-L1 was performed in a variety of biopsy samples obtained from these patients both before and during study treatment, and responses appeared to correlate, albeit incompletely, with PD-L1 expression. Lack of a universally validated antibody or assay for PD-1 and PD-L1 remains a hindrance in determining if tumor and/or accessory cell expression of PD-L1 or infiltrating PD-1-expressing lymphocytes may be used as a predictive biomarker for anti-PD-1 and PD-L1 agents. Furthermore, different IHC expression cutoff levels have been used to evaluate the predictive role of PD-L1 expression. PD-L1 expression might be dynamic, and its expression may change in an ongoing adaptive immune response. It may therefore be best used as a reflection or marker of the immune response at a particular time point to guide the choice of monotherapy or combination therapy.

To date, data for the relationship between pembrolizumab and PD-L1 expression are largely limited to data from KEYNOTE-001. In a 195-patient training set derived from the melanoma population of KEYNOTE-001 and using a 1 % cutoff to determine positivity, 71 % of the 125 evaluable patients had PD-L1-positive tumors as assessed using a prototype IHC assay and the 22C3 antibody [35]. PD-L1 positivity was associated with a higher ORR by RECIST v1.1 (49 % vs 13 %; *P* = 0.0007) and improved PFS (median PFS 11 months vs 3 months; HR = 0.52; 95 % CI: 0.32–0.86; *P* = 0.0051), but not OS (6-month OS was 91 % in positive vs 79 % in negative PD-L1 tumors; *P* = 0.3165) [35]. In an independent validation set of 216 patients with melanoma in KEYNOTE-001, 82 % of the 150 evaluable patients had PD-L1-positive tumors [36]. Similar to the training set, PD-L1 positivity vs PD-L1 negativity was associated with a higher ORR (36 % vs 4 %; *P* = 0.0022), longer PFS (HR = 0.43; 95 % CI: 0.27–0.69; *P* = 0.0002), and improved OS (HR = 0.33; 95 % CI: 0.18–0.63; *P* = 0.0042) [36]. Although PD-L1 positivity is correlated with response to pembrolizumab in patients with melanoma, given the responses seen in patients with PD-L1-negative tumors and the high prevalence of PD-L1 positivity, it is unlikely that PD-L1 will be used as a selection or predictive marker for anti-PD-1 or anti-PD-L1 agents in melanoma. Data from the registration KEYNOTE-006 trial confirm activity in PD-L1-negative tumors. However, only 20 % of the tumors were PD-L1 negative.

Results have differed in patients with lung cancer treated in KEYNOTE-001 [22]. Patients in this trial were divided (with respect to PD-L1 staining) into training (n = 182) and validation (n = 313) groups. A tumor biopsy was required 60 days prior to treatment with pembrolizumab. Initial staining was performed using the prototype assay and after a relationship was seen between degree of PD-L1 positivity and response (n = 51 patients recruited by that point), the trial was amended to include a co-primary endpoint of response in previously treated patients with high PD-L1 expression only [22]. The clinical trial assay was used for all staining thereafter using the same 22C3 antibody. The training set data were analyzed using receiver operating curves (ROC) to determine the optimal expression cutoff point. PD-L1 expression was determined as a percentage of carcinoma cell membranous staining [22]. During training analysis a critical 6-month period for retention of PD-L1 antigen in tumor sections was discovered. Validation therefore was only performed in tumor samples sectioned within 6 months of biopsy and staining. Patients, investigators and the sponsor were masked to PD-L1-staining results until at least 5 months of follow-up [22]. In total, 129 patients were used in the training set analysis and 204 (156 previously treated and 48 treatment-naive) patients were included in the validation set analysis. A cutoff of ≥50 % PD-L1 positivity in tumor cells was determined as optimal by ROC analysis [22]. Using this cutoff in the training set, ORR by RECIST v1.1 was 36.6 % (95 % CI: 22.1–53.1). In the validation group, patients with measurable disease and a score of ≥50 % staining (n = 73) had a response of 45.2 % (95 % CI: 33.5–57.3); ORR was 43.9 % (95 % CI: 30.7–57.6) in previously treated patients and 50 % (95 % CI: 24.7–75.3) in treatment-naive patients. Response was lower in patients with a score of 1 %–49 % staining (ORR 15.6 % [95 % CI: 8.3–25.6]) and ≤1 % staining (ORR 9.1 % [95 % CI: 1.1–29.2]). ORR in all patients (measurable and unmeasurable) was 42.3 % [22]. The difference in response between patients with ≥50 % staining and those with 1 %–49 % or ≤1 % staining was observed in both previously treated and treatment-naive patients. No additional differences in response rates were found according to dose, schedule, or smoking status [22]. Overall prevalence of PD-L1 staining in the screened population (n=824) using the clinical trial assay showed 23.2 % of patients to stain ≥50 % positive (24.9 % treatment naive and 22.7 % previously treated), 37.6 % to stain 1 %–49 % positive, and 39.2 % to have ≤1 % PD-L1 positivity. Only *KRAS* positivity was associated with a higher PD-L1 positivity [22]. Median duration of response was similar regardless of PD-L1 positivity, while PFS and OS were shorter in the patients with ≤1 % and 1 %–49 % PD-L1 staining compared with those with ≥50 % staining. Median PFS in the latter group was 6.3 months (95 % CI: 2.9–12.5), 6.1 months (95 % CI, 2.1-12.5) in previously treated patients and 12.5 months (95 % CI, 2.4-12.5) in treatment naive patients. Median OS was not reached in the ≥50 % PD-L1-positive group [22]. Taken together, the results suggest enrichment by PD-L1 staining for better survival outcomes in patients treated with pembrolizumb if they have ≥50 % PD-L1-tumor positivity. Whether PD-L1 is a prognostic marker is yet to be determined, although a meta-analysis has suggested that it does not have a positive effect in NSCLC cancer [37]. Given the differences in assays and cutoff levels used to define PD-L1 positivity, further data and longer follow-up are required to evaluate its use as a prognostic marker.

Although its use in lung cancer is not fully defined, the ongoing KEYNOTE-010, KEYNOTE-024, and KEYNOTE-042 studies of pembrolizumab are limiting enrollment to patients with PD-L1-positive tumors. Interestingly PD-L1 expression has also been shown to be a predictor of response to MPDL3280A (a PD-L1 antibody), but with expression on tumor-infiltrating immune cells more indicative of a response than expression on tumor cells [38].

A number of other predictive biomarkers of response to pembrolizumab have been proposed. Analysis of the sum of target lesions at baseline (“baseline tumor size”) in patients with melanoma enrolled in KEYNOTE-001 showed that baseline tumor size below vs above the median was found to independently predict both response (ORR 42 % vs 25 %; *P* = 0.001) and OS (HR = 2.35; *P* < 0.001), although all patients could benefit from treatment [39]. Baseline tumor size remained an independent predictor of response and OS in an extended analysis that included PD-L1 [40]. Infiltration of tumor by CD8^+^ T cells at the invasive tumor margin and expression of neoepitopes have both been described as additional predictors of response to pembrolizumab in melanoma and NSCLC, respectively [41, 42].

### An overview of and perspective on therapeutic targeting of the PD-1/PD-L1 axis

Several agents targeting the PD-1/PD-L1 axis are in development, including the PD-1 inhibitors nivolumab, AMP-224, and pidilizumab and the PD-L1 inhibitors BMS-936559, MPDL3280A, and MEDI 4736 (Table 4). Nivolumab and pembrolizumab are the 2 most advanced agents in terms of clinical development. Pembrolizumab alone is being studied in ≥20 tumor types. Given the single-agent activity seen to date with all of these agents, it remains unclear whether novel combinations will provide acceptable tolerability and greater efficacy. Nivolumab in combination with ipilimumab is being evaluated in a number of tumor types, including melanoma. Phase II data from the CheckMate 069 double-blinded trial of nivolumab and ipilimumab vs ipilimumab alone have shown an objective response rate of 61 % for the combination vs 11 % for ipilimumab and placebo (*P* < 0.001) [43]. The primary endpoint of this trial was ORR among patients with *BRAF*^V600^ wild-type tumors. Mature data on PFS (secondary endpoint) are awaited. Data from the registration phase III CheckMate 067 trial (double-blind trial of nivolumab vs ipilimumab vs combination of these agents (ClinicalTrials.gov identifier: NCT01844505) are eagerly awaited. Pembrolizumab is also being studied in combination with ipilimumab in patients with melanoma and RCC in the phase I/II KEYNOTE-029 study (ClinicalTrials.gov identifier: NCT02089685). Other novel combinations include nivolumab in combination with the checkpoint inhibitor LAG-3 (ClinicalTrials.gov identifier: NCT01968109) and pembrolizumab plus the IDO1 inhibitor INCB024360 in KEYNOTE-037 (ClinicalTrials.gov identifier: NCT02178722). Addition of stimulatory CD137 agonists is being evaluated in studies of nivolumab plus urelumab (NCT02253992), and pembrolizumab plus PF-05082566, a 4-1BB ligand inhibitor (NCT02179918). Pembrolizumab is also being studied in combination with pegylated interferon alpha-2b, talimogene laherparepvec, dabrafenib, trametinib, axitinib, pazopanib, chemotherapy, cetuximab, trastuzumab, and ADXS31-142.

**Table 4:** Overview of PD-1/PD-L1 inhibitors with active clinical trials

Compound	Developing company	Most advanced stage of development by cancer type
PD-1		
Pembrolizumab (MK-3475, SCH 900475)	Merck & Co.	• Approved (various countries): advanced melanoma
		• Phase III: head and neck cancer; melanoma; NSCLC; urothelial cancer; gastric/gastroesophageal junction adenocarcinoma
		• Phase II: colorectal cancer; Merkel cell cancer; multiple myeloma; mycosis fungoides or Sezary syndrome; prostate cancer; soft tissue sarcoma; bone sarcoma; GBM; mesothelioma; Hodgkin lymphoma; non-Hodgkin lymphoma (DLBCL); breast cancer; small cell lung cancer; bladder cancer; thymic carcinoma; renal cell cancer; pancreatic cancer; chronic lymphocytic leukemia
		• Phase I: advanced solid tumors; myelodysplastic syndrome; pontine gliomas; ovarian cancer
Nivolumab (BMS-936558, MDX1106, ONO-4538)	Bristol-Myers Squibb/Ono Pharmaceutical	• Approved (various countries): advanced melanoma; previously treated squamous cell NSCLC
		• Phase III: gastric cancer; GBM; head and neck cancer; melanoma; NSCLC; renal cell carcinoma
		• Phase II: acute myeloid leukemia; anal carcinoma; B-cell non-Hodgkin lymphoma; cervical cancer; follicular lymphoma; Hodgkin lymphoma; nasopharyngeal carcinoma; chronic lymphocytic leukemia; pancreatic adenocarcinoma; urothelial cancer; myelofibrosis
		• Phase I: advanced solid tumors; breast cancer; chronic myeloid leukemia; colorectal cancer; DLBCL; Ewing sarcoma; hepatocellular carcinoma; multiple myeloma; osteosarcoma; ovarian cancer; rhabdomyosarcoma
Pidilizumab (CT-011)	Curetech	• Phase II: acute myelogenous leukemia; follicular lymphoma; multiple myeloma; pancreatic cancer; prostate cancer; renal cell carcinoma
		• Phase I: glioma
AMP-224	Amplimmune/ GlaxoSmithKline	• Phase I: colorectal cancer
AMP-514 (MEDI0680)	Amplimmune	• Phase I: advanced malignancies; B-cell lymphomas
REGN2810	Regeneron	• Phase I: advanced malignancies
PD-L1		
MPDL3280A	Genentech/Roche	• Phase III: NSCLC; urothelial cancer; renal cell carcinoma; breast cancer
		• Phase II: colorectal cancer
		• Phase I: advanced solid tumors; DLBCL; follicular lymphoma; melanoma
MEDI4736	Medimmune/AstraZeneca	• Phase III: NSCLC; head and neck cancer
		• Phase II: colorectal cancer; GBM
		• Phase I: advanced solid tumors; B-cell lymphoma; cervical cancer; gastric/gastroesophageal junction cancer; melanoma; myelodysplastic syndrome; breast cancer; pancreatic cancer
BMS-936559 (MDX-1105)	Bristol-Myers Squibb	• Phase I: advanced solid tumors
MSB0010718C	Merck Serono	• Phase III: NSCLC
		• Phase II: Merkel cell carcinoma
		• Phase I: advanced solid tumors

Information derived from ClinicalTrials.gov (access date: April 28, 2015)

Abbreviations: *DLBCL* diffuse large B-cell lymphoma; *GBM* glioblastoma multiforme; *NSCLC* non-small cell lung cancer; *PD-1* programmed death receptor 1; *PD-L1* programmed death receptor ligand 1

## Conclusions

The development of pembrolizumab from the first-in-humans study to FDA approval for the treatment of patients with unresectable or metastatic melanoma with disease progression following ipilimumab, and if *BRAF*^V600^ mutation positive, a BRAF inhibitor, has occurred in a record 3.6 years. Approval is likely to be extended to include other melanoma populations and other tumor types. The dose of pembrolizumab currently approved in metastatic melanoma is 2 mg/kg Q3W. Higher doses are being explored in other tumor types but there have been no statistically significant differences in responses or outcomes with 10 mg/kg on either Q2W or Q3W schedules in KEYNOTE-001 cohorts. It is likely that the Q3W schedule will therefore be chosen. Future trials are evaluating a fixed dose of 200 mg in a variety of carcinomas. Furthermore, mature data are needed on patterns and kinetics of response and toxicity. The optimum duration of treatment is unknown, as is the risk of late serious or treatment-limiting toxicity especially in the context of CR or near CR. Patients in KEYNOTE-001, KEYNOTE-002, and KEYNOTE-006 who had a confirmed complete response were allowed to discontinue treatment and enter follow-up. Mature data on outcomes in these patients specifically and dedicated trials of different durations of treatment will inform on this question.

Despite the clinical urgency to develop these agents, well-thought-out translational research will be crucial in determining predictive biomarkers and elucidating mechanisms of resistance to plan rational therapeutic combinations. For example, infiltration of CD8^+^ T-cells, PD-L1 staining, and the presence of other checkpoint inhibitors may aid treatment choices by informing the use of combination therapy vs monotherapy. Intrapatient heterogeneity in response may be further combated by adjunctive therapies such as surgery or radiotherapy to induce an abscopal effect. Taken together, the approval of pembrolizumab as a first-in-class PD-1 inhibitor has been a defining moment in immuno-oncology and promises to accelerate the field for decades to come.

## Abbreviations

AE: Adverse event
ALK: Anaplastic lymphoma kinase
ALT: Alanine aminotransferase
APC: Antigen-presenting cell
CI: Confidence interval
CR: Complete response
DLBCL: Diffuse large B-cell lymphoma
DR: Drug-related
DRAE: Drug-related adverse event
EGFR: Epidermal growth factor receptor
FDA: US Food and Drug Administration
GBM: Glioblastoma multiforme
HNSCC: Head and neck squamous cell carcinoma
HPV: Human papilloma virus
HR: Hazard ratio
IFN-γ: Interferon gamma
IHC: Immunohistochemical
irRC: Immune-related response criteria
LDH: Lactate dehydrogenase
MRI: Magnetic resonance imaging
NR: Not reached/not reported
NSCLC: Non-small cell lung cancer
ORR: Overall response rate
OS: Overall survival
PD-1: Programmed death receptor 1
PD-L1: Programmed death receptor ligand 1
PD-L2: Programmed death receptor ligand 2
PFS: Progression-free survival
PR: Partial response
Q2W: Once every 2 weeks
Q3W: Once every 3 weeks
RECIST v1.1: Response Evaluation Criteria in Solid Tumors version 1.1
ROC: Receiver operating curve
SD: Stable disease
TNF: Tumor necrosis factor
